# Exosomes Derived from Human Induced Pluripotent Stem Cells Ameliorate the Aging of Skin Fibroblasts

**DOI:** 10.3390/ijms19061715

**Published:** 2018-06-09

**Authors:** Myeongsik Oh, Jinhee Lee, Yu Jin Kim, Won Jong Rhee, Ju Hyun Park

**Affiliations:** 1 Department of Medical Biomaterials Engineering, Kangwon National University, 1 Kangwondaehak-gil, Chuncheon-si, Gangwon-do 24341, Korea; okm934@naver.com (M.O.); mellowuu92@gmail.com (Y.J.K.); 2 Division of Bioengineering, Incheon National University, Incheon 22012, Korea; leehee8819@gmail.com (J.L.); wjrhee@inu.ac.kr (W.J.R.); 3 Institute of Bioscience and Biotechnology, Kangwon National University, 1 Kangwondaehak-gil, Chuncheon-si, Gangwon-do 24341, Korea

**Keywords:** human induced pluripotent stem cells (iPSCs), exosomes, skin regeneration, photoaging, senescence

## Abstract

Stem cells and their paracrine factors have emerged as a resource for regenerative medicine. Many studies have shown the beneficial effects of paracrine factors secreted from adult stem cells, such as exosomes, on skin aging. However, to date, few reports have demonstrated the use of exosomes derived from human pluripotent stem cells for the treatment of skin aging. In this study, we collected exosomes from the conditioned medium of human induced pluripotent stem cells (iPSCs) and investigated the effect on aged human dermal fibroblasts (HDFs). Cell proliferation and viability were determined by an MTT assay and cell migration capacity was shown by a scratch wound assay and a transwell migration assay. To induce photoaging and natural senescence, HDFs were irradiated by UVB (315 nm) and subcultured for over 30 passages, respectively. The expression level of certain mRNAs was evaluated by quantitative real-time PCR (qPCR). Senescence-associated-β-galactosidase (SA-β-Gal) activity was assessed as a marker of natural senescence. As a result, we found that exosomes derived from human iPSCs (iPSCs-Exo) stimulated the proliferation and migration of HDFs under normal conditions. Pretreatment with iPSCs-Exo inhibited the damages of HDFs and overexpression of matrix-degrading enzymes (MMP-1/3) caused by UVB irradiation. The iPSCs-Exo also increased the expression level of collagen type I in the photo-aged HDFs. In addition, we demonstrated that iPSCs-Exo significantly reduced the expression level of SA-β-Gal and MMP-1/3 and restored the collagen type I expression in senescent HDFs. Taken together, it is anticipated that these results suggest a therapeutic potential of iPSCs-Exo for the treatment of skin aging.

## 1. Introduction

Skin undergoes physiological changes as a consequence of the aging process. There are two basic types of skin aging, i.e., intrinsic and extrinsic aging. Intrinsic aging is genetically determined, which indicates that it occurs inevitably as time passes. Many studies have suggested epigenetic changes and post-translational mechanisms are more important pathways of intrinsic aging rather than genetic influence. On the other hand, extrinsic aging occurs by external factors such as smoking, air pollution, and unbalanced nutrition. Among them, UV exposure is the most important cause of extrinsic aging. Therefore, the skin damages induced by UV exposure is called “photoaging” [1,2]. Photoaging is characterized by irregular pigmentation, dryness, sallowness, roughness, premalignant lesions, and skin cancer [3]. Intrinsic skin aging, in contrast, is characterized by a loss of elasticity and fine wrinkles rather than deep wrinkles due to photoaging [4,5].

The dermis is a layer of skin that lies between the epidermis and the subcutaneous layer. Structural components of the skin dermis are the collagen fibril, elastic fibers, and an extrafibrillar matrix composed of proteoglycans, glycosaminoglycans, and several glycoproteins [6,7]. Fibroblasts are the primary cell types constituting the dermis and are responsible for the synthesis of structural components such as procollagen and elastic fibers. Fibroblasts lose their capacities for proliferation and synthesis of collagen, the major extracellular matrix constituent of the skin dermis, with aging [8,9]. On the other hand, the expression of various types of matrix-degrading metalloproteinase (MMP) is upregulated in the aged fibroblasts [10]. Age changes the number and proliferation of dermal fibroblasts, reduces collagen synthesis and repair, and accelerates degradation of the existing skin matrix by MMPs, thereby reducing the regenerative capacity of skin. Many studies have shown that the age-related reductions in proliferation and collagen synthesis of fibroblasts are linked with the downregulation of growth-associated factors such as platelet-derived growth factor (PDGF) and the transforming growth factor-β (TGF-β) [4,11]. PDGF is a growth-stimulating mitogen for fibroblasts. Binding of PDGF to specific receptors results in mitogen-activated protein kinase (MAPK) signaling, which induces cell proliferation [12]. TGF-β plays a crucial role in matrix biosynthesis of dermal fibroblasts. Many extracellular matrix (ECM)-related genes, such as various types of collagens and fibronectins, were upregulated and MMP genes were downregulated by TGF-β signaling [13,14]. Cellular responsiveness to PDGF and TGF-β is reduced in aged fibroblasts because the expression levels of their major receptors located in the plasma membrane were reduced with aging [11]. Reductions in the proliferation of fibroblasts and regeneration of ECM molecules accelerate the breakdown of connective tissue, which results in external skin aging, such as wrinkles [15].

Stem cells have been widely used for skin regeneration. Recently, it has been demonstrated in several preclinical and clinical studies that the transplantation of mesenchymal stem cells (MSCs) contributes to wound repair and regeneration [16,17,18]. However, paracrine actions of the transplanted stem cells are believed to play a crucial role in the therapeutic effects, as well as the alteration of injured tissues by transdifferentiation [19,20]. Many studies have reported that stem cells secrete several cytokines such as TGF-β, PDGF, and basic fibroblast growth factor, which promote the proliferation of dermal fibroblasts and the synthesis of ECM molecules. Huh et al. mentioned that the conditioned medium (CM) of human amniotic fluid-derived stem cells improved skin regeneration after UVA-induced photoaging by accelerating the proliferation and migration of dermal fibroblasts [21]. Li et al. reported the restoration effects of human chorion-derived stem cell CM on photoaged human epidermal cells [22]. In addition, Shim et al. revealed the beneficial effects of human dermal stem/progenitor cells (hDSPCs)-derived CM on UVA-induced damages of human dermal fibroblasts (HDFs) [23]. They also demonstrated that the hDSPCs-CM reduced the production of senescence-associated β-galactosidase (SA-β-Gal) and restored the changes in the expression level of MMP-1, tissue inhibitor of metalloproteinase, and collagen I/III in naturally senescent HDFs [24].

In addition, there have been advances in exploring the roles of exosomes secreted from stem cells in these paracrine actions. Exosomes are small membrane lipid vesicles secreted by most cell types (30–120 nm in diameter) [25,26]. The formation of exosomes is associated with the endosomal network, which sorts the various transport vesicles. Early endosomes undergo a series of maturation steps to form late endosomes, also known as multivesicular bodies (MVBs). When the late endosomes fuse with the plasma membrane, small-sized vesicles are released out of the cell, which are the exosomes [27]. Exosomes contain functional messenger RNAs (mRNAs) and micro RNAs (miRNAs), as well as several proteins, that originate from the host cells [28]. Several evidences have also been revealed that the presence of several classes of long noncoding RNAs (lncRNAs) in exosomes [29]. As lncRNAs have the function to induce epigenetic modifications by binding to specific genomic loci and recruiting epigenetic regulators such as chromatin remodeling complexes, exosomes secreted from one cell may also induce epigenetic modifications in recipient cells. Therefore, exosomes are considered to be implicated in cell-to-cell communication and the progression of several diseases such as cancer [30,31]. Recently, many studies have demonstrated the therapeutic applications of exosomes derived stem cells to ameliorate various injuries, including cardiovascular, renal, and lung injuries [32,33].

We previously demonstrated the stimulatory effects of human induced pluripotent stem cell-conditioned medium (iPSC-CM) on the proliferation and migration of dermal fibroblasts [34]. Herein, we hypothesized that the iPSCs-CM contained exosomes and the human induced pluripotent stem cells-derived exosomes (iPSC-Exo) played a key role in these effects of iPSC-CM. To address this hypothesis, we isolated exosomes from iPSC-CM and examined their effects on several cellular responses associated with skin aging, as well as the proliferation and migration in HDFs. To induce photoaging and natural senescence, HDFs were irradiated by UVB or subcultured and serially passaged, respectively. Then, we explored the protective effect of iPSC-Exo on UVB irradiation-induced cellular damage. Furthermore, we examined the effect of iPSC-Exo on the expression of MMP-1, MMP-3, and collagen type I, which are important in the construction of ECM, in UVB-induced and naturally senescent HDFs, respectively.

## 2. Results

### 2.1. Characterization of Exosomes Derived from Human iPSCs

**Figure 1:**
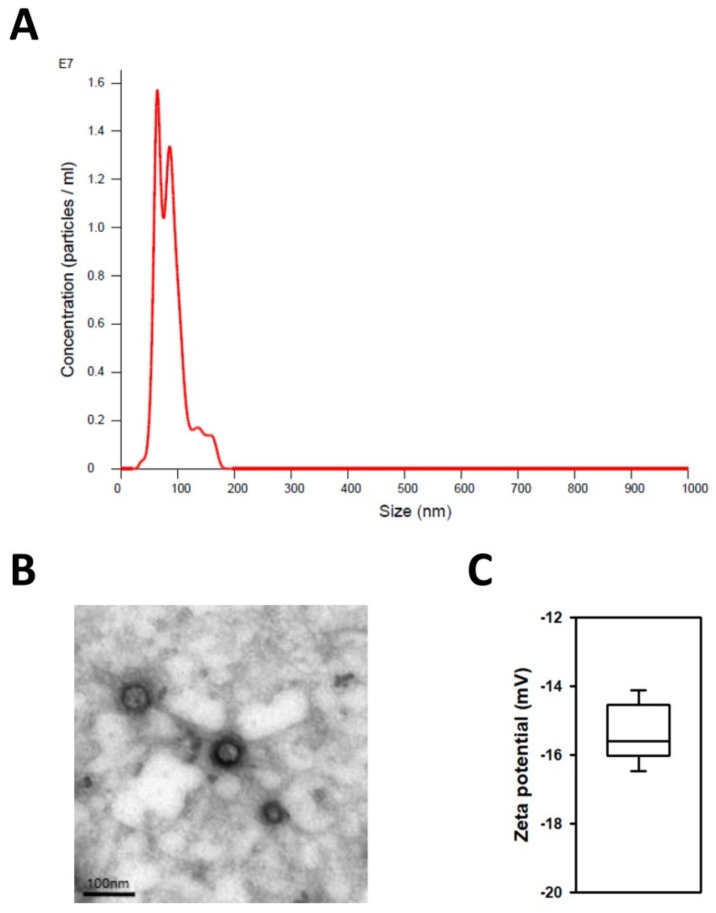
Characterization of iPSC-Exo. (**A**) Nanoparticle tracking analysis (NTA) result of iPSC-Exo. Mean diameter of iPSC-Exo was 85.8 nm. (**B**) The transmission electron microscopy (TEM) image of iPSC-Exo morphology (scale bar = 100 nm). (**C**) Dynamic light scattering (DLS) data for iPSC-Exo. The mean value for zeta potential of iPSC-Exo was −15.6 mV.

Exosomes secreted from human iPSCs were isolated using ExoQuick-TC^TM^ solution (System Biosciences, Palo Alto, CA, USA) as described in the experimental section and characterized by size and morphology. We utilized nanoparticle tracking analysis (NTA) to evaluate exosome numbers and their size profiles. The NTA results showed that the size distribution of iPSC-Exo had a major peak at ~100 nm and the mean diameter was 85.8 nm, although a small number of larger vesicles was included (Figure 1A). This result indicated that most of the extracellular vesicles used in this study were exosomes, because the sizes of other microvesicles or apoptotic bodies are relatively larger than exosomes. Transmission electron microscopy (TEM) images indicated a similar tendency as the NTA result, showing that iPSC-Exo had a spherical membrane structure (Figure 1B). The sizes of exosomes shown in the TEM image were smaller than that from the NTA result. It is believed that this is due to condensation of the iPSC-Exo during the drying step for TEM analysis [35]. In addition, dynamic light scattering (DLS) results showed that the average zeta potential of the isolated iPSC-Exo was −15.6 mV (Figure 1C). These results suggest that the physical characteristics of iPSC-Exo used in this study were similar to those of exosomes derived from other cell lines [36,37].

### 2.2. Proliferation and Migration of HDFs Were Accelerated by iPSC-Exo

**Figure 2:**
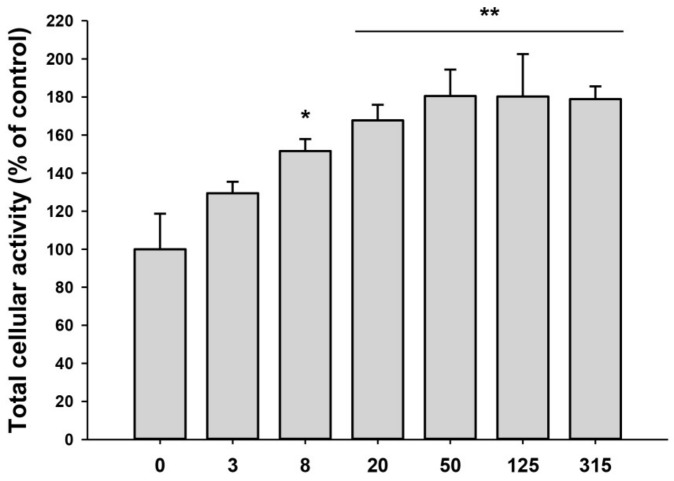
Effect of iPSC-Exo on the proliferation of human dermal fibroblasts (HDFs). Serum-starved HDFs were treated with iPSC-Exo for 48 h. The population of live HDFs was determined by 3-(4,5-dimethylthiazol-2-yl)-2,5-diphenyltetrazolium bromide (MTT) assay. The total population of the iPSC-Exo-untreated group was regarded as 100%, and the populations of other groups were estimated as relative values. * *p* < 0.05, ** *p* < 0.01 compared to the iPSC-Exo-untreated group. Error bars indicate standard deviations of triplicate samples in a single representative experiment.

**Figure 3:**
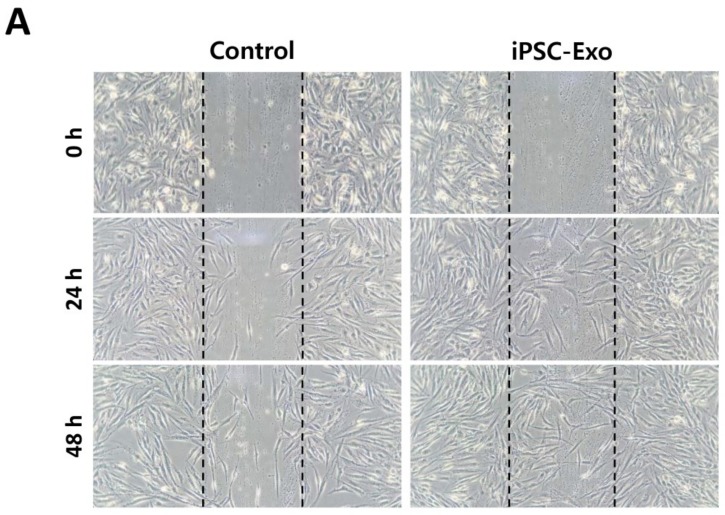

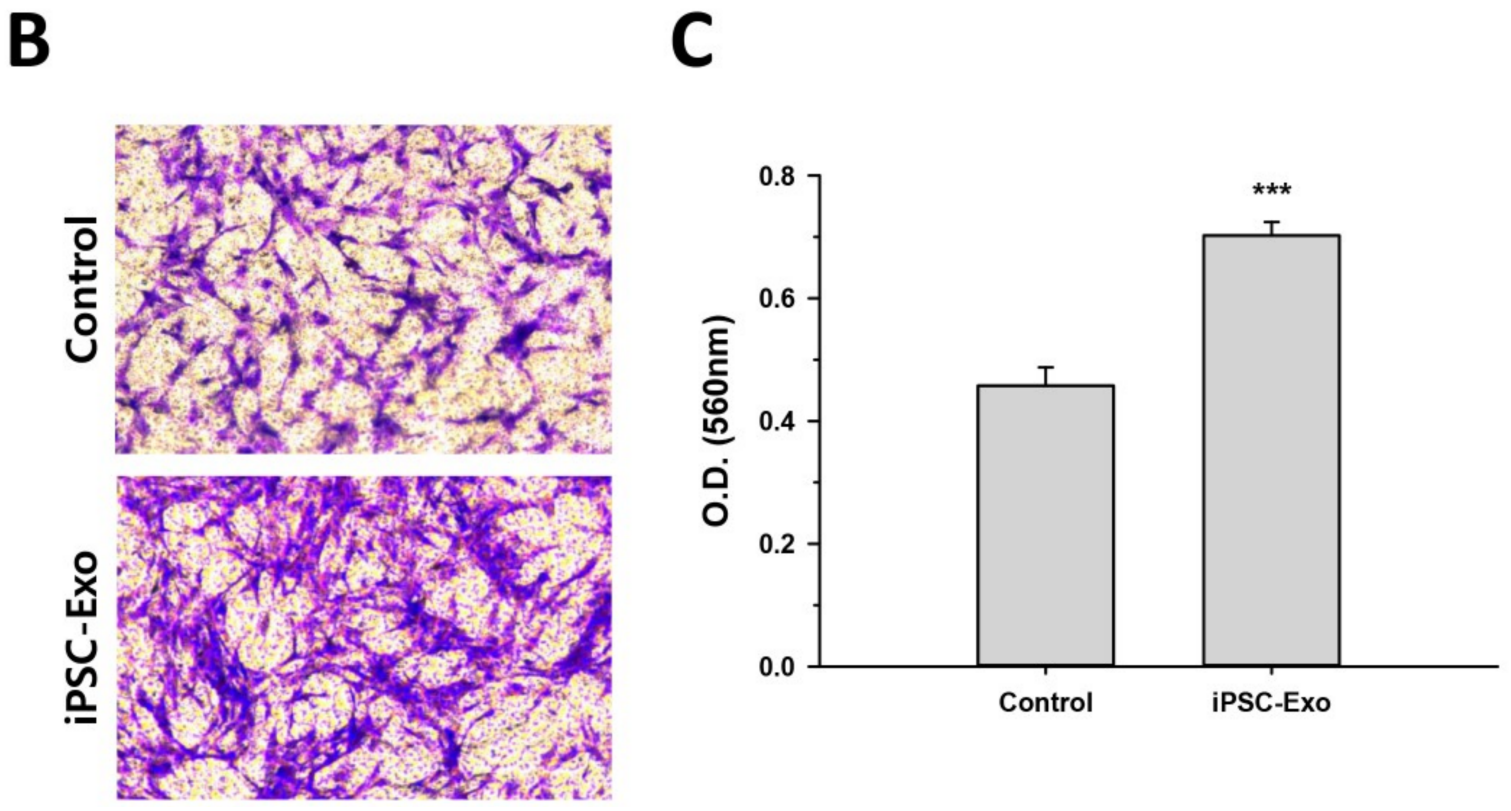
iPSC-Exo promoted the migration of HDFs. (**A**) Scratch wound assay for HDFs treated with iPSC-Exo. Serum-starved HDFs were scratched and simultaneously treated with 20 × 10^8^ particles/mL iPSC-Exo. The representative image indicates more rapid migration of the iPSC-Exo-treated HDFs into the scratched area. (**B**) Representative image of a transwell assay for migration of HDFs. HDFs attached to the upper side of the transwell membrane migrated to the lower side in serum-free medium without or with iPSC-Exo for 24 h. The cells attached to the bottom of the membrane were fixed with 4% paraformaldehyde and stained with crystal violet. (**C**) Quantitative analysis of a transwell assay. Transwell membrane with the stained HDFs was cut out and immersed in a 50% acetic acid solution to dissolve the crystal violet. The amount of stained crystal violet was determined as absorbance at 560 nm. *** *p* < 0.001 compared to the iPSC-Exo-untreated group. Error bars indicate standard deviations of triplicate samples in a single representative experiment.

We previously demonstrated that the iPSC-CM could promote proliferation and migration of HDFs. As the iPSC-CM was supposed to contain exosomes, we examined the effect of iPSC-Exo on proliferation and migration of HDFs. The mitogenic effect was determined by the MTT assay with different doses of iPSC-Exo, ranging from 3 to 315 × 10^8^ particles/mL. The proliferation of HDFs was increased by iPSC-Exo in a dose-dependent manner up to 50 × 10^8^ particles/mL and maintained with a higher concentration (Figure 2). In our previous study, some toxicity had been shown at high concentrations of iPSC-CM [34]. However, no toxic effect was observed when iPSC-Exo was treated even with higher concentrations. Although the mitogenic effect was saturated up to 50 × 10^8^ particles/mL, we used iPSC-Exo with 20 × 10^8^ particles/mL in further experiments because the differences were not significant. To investigate the effect of iPSC-Exo on migration of HDFs, we performed scratch wound assays. The stimulatory effect on cell migration was determined by evaluating the closure of the scratched area in this assay. As shown in Figure 3A, the rates of gap-filling in HDFs treated with iPSC-Exo were significantly higher than the control group, indicating that iPSC-Exo promoted the migration of HDFs. The migration-promoting effect was also demonstrated in a transwell migration assay. When the cells were seeded on the upper side of a porous membrane, they migrated to the opposite side across the membrane. HDFs that migrated toward the bottom side of the membrane were stained by crystal violet, and the intracellular crystal violet was quantitated by measuring the absorbance at 560 nm after dissolving it with an acetic acid solution. Bright field images showed that the number of migrated cells was significantly increased by the treatment of iPSC-Exo (Figure 3B). The result of the quantitative analysis also demonstrated the stimulatory effect of iPSC-Exo on the migration of HDFs (Figure 3C).

### 2.3. Effect of iPSC-Exo on UVB-Induced HDFs Damages

**Figure 4:**
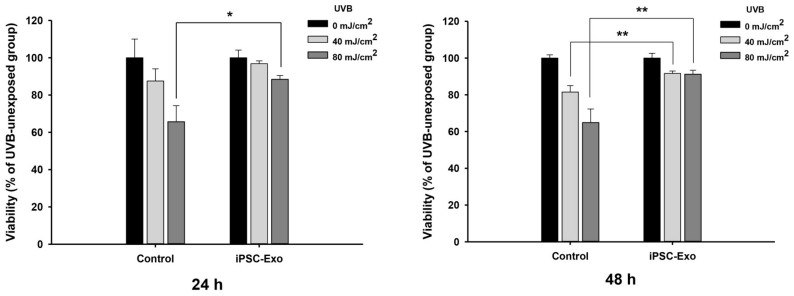
Protective effect of iPSC-Exo on UVB-induced cell damage. HDFs were treated with 20 × 10^8^ particles/mL iPSC-Exo for 24 h in serum-free DMEM/F12 and simultaneously irradiated with UVB (315 nm). After further incubation for the indicated time interval in serum-containing growth medium, the populations of viable cells were determined by MTT assay. * *p* < 0.05, ** *p* < 0.01 compared to the UVB-irradiated and iPSC-Exo-untreated group. Error bars indicate standard deviations of triplicate samples in a single representative experiment.

To investigate whether iPSC-Exo can rescue HDFs from the damage induced by UVB irradiation, cells were treated with 20 × 10^8^ particles/mL of iPSC-Exo prior to UVB irradiation. Since it will take some time for iPSC-Exo to pass through the plasma membrane and mediate cellular signaling pathways caused by UVB irradiation, it was considered that the treatment of iPSC-Exo after UVB irradiation would not show significant recuperative effects. When the cells were irradiated by 40 mJ/cm^2^ UVB, the difference was not statistically significant (0.05 < *p* < 0.1) 24 h after UVB irradiation, although the iPSC-Exo-treated group showed slightly higher viabilities. However, when the cells were irradiated by 80 mJ/cm^2^ UVB, the viabilities of the untreated group were significantly reduced to 65.6% ± 8.6%, while the iPSC-Exo-treated group showed viabilities of 88.4% ± 2.1% (Figure 4). After 48 h of UVB irradiation, the effect of iPSC-Exo on UVB-induced cell damage was more clearly demonstrated when the cells were irradiated, with 40 mJ/cm^2^ as well as 80 mJ/cm^2^ UVB. These results indicate that iPSC-Exo can protect human skin cells from UV-induced photoaging.

### 2.4. Effect of iPSC-Exo on the Expression of MMP-1, MMP-3, and Collagen Type I at mRNA Level

**Figure 5:**
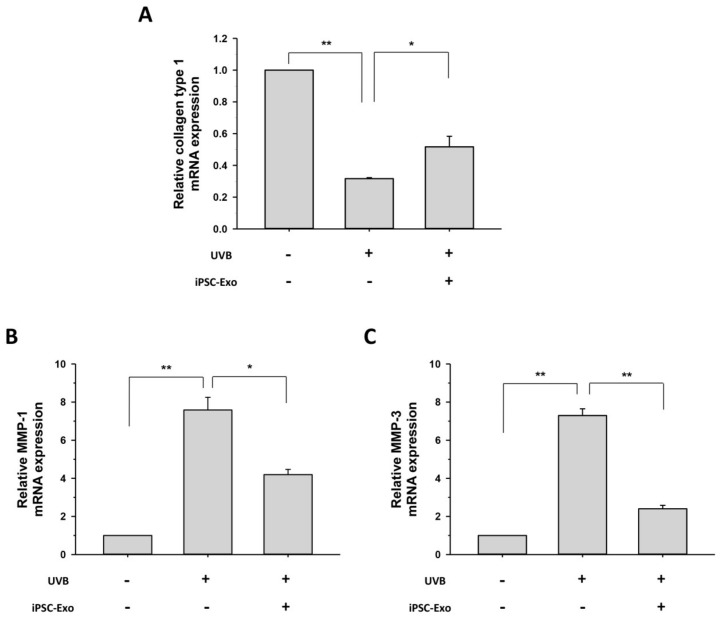
iPSC-Exo restored the altered expression of specific dermal markers in UVB-irradiated HDFs. After treatment with 20 × 10^8^ particles/mL iPSC-Exo for 24 h in serum-free DMEM/F12, HDFs were harvested after 48 h. The mRNA expression levels of collagen type I (**A**); MMP-1 (**B**); and MMP-3 (**C**) were quantified by quantitative real-time RT-PCR. * *p* < 0.05, ** *p* < 0.01. Error bars indicate standard deviations of triplicate samples in a single representative experiment.

Collagen type I predominates in the dermis and is responsible for the tensile strength of the skin tissue. MMPs are a family of the structurally related matrix-degrading enzymes. It has been reported that UV irradiation inhibits collagen type I synthesis and induces the expression of MMPs, which eventually results in premature skin aging [10,38,39]. As UVB irradiation has been reported to alter the expression of a wide range of genes through the regulation of several transcription factors [39,40], we investigated the effect of iPSC-Exo on mRNA expression of genes related to photoaging. After UVB irradiation of HDFs, the levels of MMP-1, MMP-3, and collagen type I mRNA were evaluated using quantitative real-time PCR (qPCR). UVB irradiation (80 mJ/cm^2^) increased the mRNA expression of MMP-1 and MMP-3 by 7.6 ± 0.7 and 7.3 ± 0.3 times, respectively. On the other hand, the mRNA expression of collagen type I was reduced by 68.3% ± 0.5% by UVB irradiation. However, it was clearly demonstrated that iPSC-Exo treatment significantly increased transcript levels of collagen type I, which is a crucial component of the skin dermis (Figure 5A). On the contrary, the expression levels of matrix-degrading enzymes, MMP-1 and MMP-3, were reduced in the HDFs treated with iPSC-Exo compared to the untreated group (Figure 5B,C). These results indicate that iPSC-Exo restored the mRNA expression disturbed by UV irradiation in the dermal fibroblasts.

### 2.5. iPSC-Exo Reversed Senescence-Related Gene Expressions in HDFs

**Figure 6:**
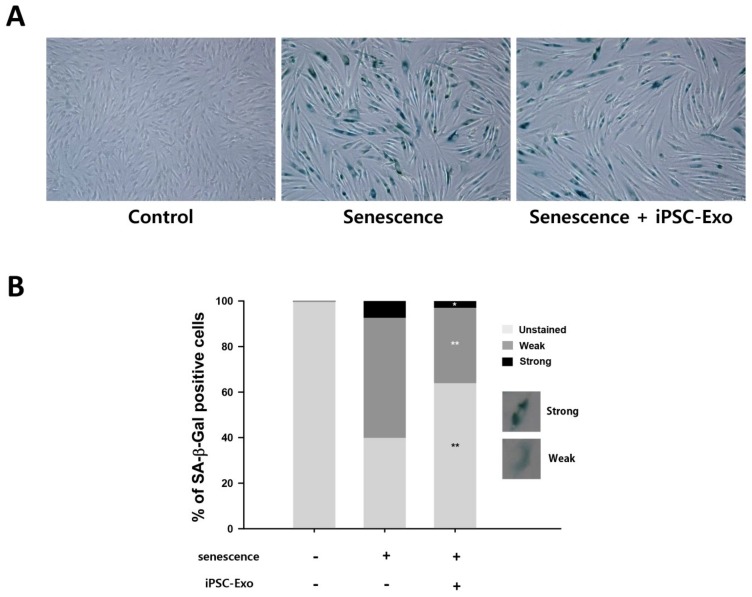
Effect of iPSC-Exo on the expression of SA-β-Gal in senescent HDFs. The passage numbers of control and senescent HDFs were 5 and 31, respectively. (**A**) SA-β-Gal-positive cells were shown in blue when observed under optical microscopy. (**B**) According to the standard shown in the figure, cells were classified into three categories: unstained, weak, and strong, and the number of cells in each group was expressed as a percentage. * *p* < 0.05, ** *p* < 0.01 compared to the iPSC-Exo-untreated senescent group. Statistical analysis was performed with three different images.

**Figure 7:**
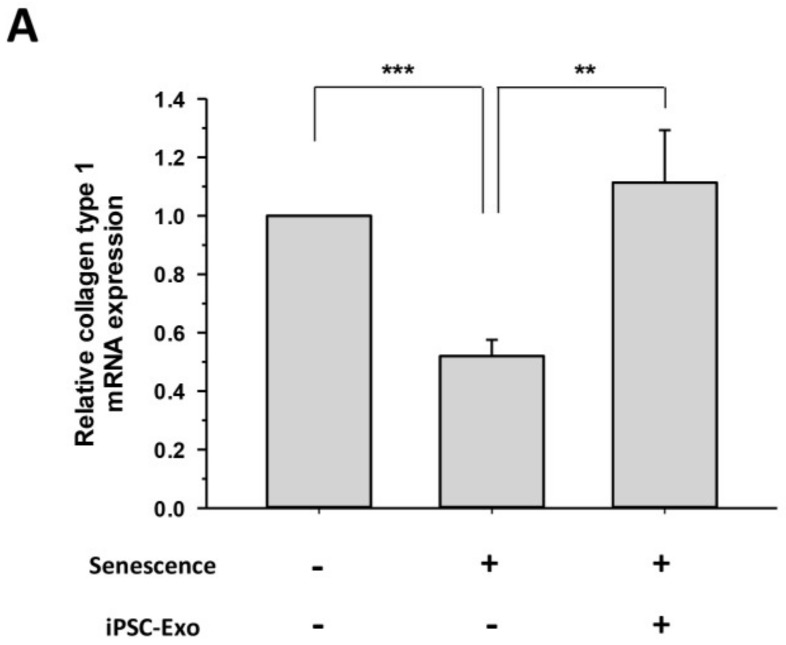

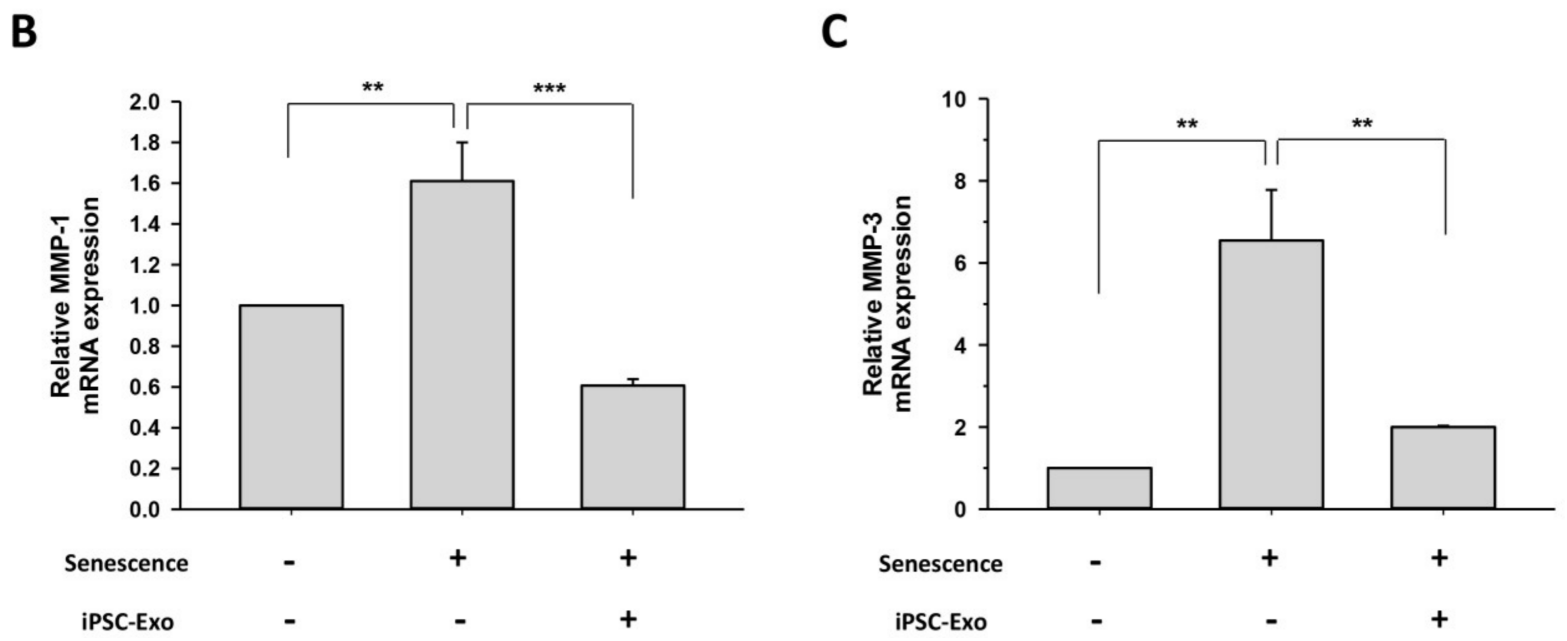
iPSC-Exo restored the altered expression of specific dermal markers in senescent HDFs. After treatment with 20 × 10^8^ particles/mL iPSC-Exo for 24 h in serum-free DMEM/F12, HDFs were harvested after 48 h. The mRNA expression levels of collagen type I (**A**); MMP-1 (**B**); and MMP-3 (**C**) were quantified by quantitative real-time RT-PCR. ** *p* < 0.01, *** *p* < 0.001. Error bars indicate standard deviations of triplicate samples in a single representative experiment.

We examined whether iPSC-Exo could restore alterations of gene expression even in naturally senescent HDFs. SA-β-Gal is a typical biomarker expressed in senescent fibroblasts. It has been demonstrated that the expression level of SA-β-Gal increases with age in epidermal keratinocytes and dermal fibroblasts of human skin biopsies [41]. Many studies have examined the expression of SA-β-Gal by staining senescent cells with a substrate at pH 6.0 [42,43]. Figure 6A shows that the expression of SA-β-Gal was higher in senescent HDFs (passage number 31) compared to young HDFs (passage number 5). However, the treatment of iPSC-Exo significantly reduced the number of SA-β-Gal-positive cells. For quantitative analysis, the cells were classified into three groups: unstained, weakly stained, and strongly stained, according to the expression of SA-β-Gal, and then the number of cells in each group was counted from three different images. As a result, it was clearly demonstrated that the proportion of SA-β-Gal-positive cells was reduced in the cells treated with iPSC-Exo compared to the untreated senescent cells (Figure 6B). Next, we investigated the effect of iPSC-Exo on gene expression associated with ECM construction in senescent HDFs. As in the case of photoaging, the mRNA levels of MMP-1 and MMP-3 were increased, and iPSC-Exo significantly reduced the expression of these matrix-degrading enzymes. On the contrary, collagen type I mRNA was reduced in senescent HDFs, and it was completely recovered through treatment of iPSC-Exo (Figure 7).

## 3. Discussion

Several factors are involved in skin aging, such as extrinsic factors, e.g., UV light and air pollution, as well as intrinsic factors, such as natural senescence. Tissue integrity is disrupted in the aging skin, resulting in the reduction of elasticity and the formation of wrinkles [44]. Many studies have demonstrated that stem cells and their paracrine factors are potential therapeutics for the treatment of several diseases, and that exosomes play an important role in their functions [19,45,46]. In recent years, there have been advances in exploring the role of exosomes secreted from stem cells in tissue regeneration. Many cytokines, RNAs, and some proteins that can induce repair following cellular injury, are located within the lipid-bilayer membranes of exosomes [28]. For this reason, exosomes are believed to have many advantages, such as reliably preserving the useful components, and delivering them into the cells in the injured tissues. Zhang et al. mentioned that exosomes of human iPSC-derived MSCs facilitate cutaneous wound healing by inducing collagen synthesis and angiogenesis [47]. Hu et al. demonstrated through in vivo tracking experiments that exosomes derived human adipose MSCs accelerate cutaneous wound healing [37]. Furthermore, Nong et al. reported the hepatoprotective effect of exosomes originating from human iPSC-derived mesenchymal stromal cells against hepatic ischemia-reperfusion injury [48]. On the other hand, some studies have revealed the therapeutic effects of human pluripotent stem cells-derived exosomes. Khan et al. reported the effect of mouse embryonic stem cell-derived exosomes for cardiac regeneration in ischemic myocardium [49]. Park and colleagues generated exosome-like nanovesicles from human embryonic stem cells through devices that physically impacts the cells. They demonstrated that the generated nanovesicles were able to enter mouse skin fibroblasts and promote cell proliferation [50]. However, according to our finding, therapeutic effects of exosomes derived from human pluripotent stem cells on the aging of skin cells as well as an in vivo study have not been reported.

We purified exosomes from the medium conditioned by human iPSCs and characterized iPSC-Exo as having spherical morphologies with approximate diameters ranging from 30 to 120 nm; these features were like the exosomes derived from other cell lines (Figure 1). In the previous report, when the iPSC-CM was treated to HDFs, it has been demonstrated that the cell proliferation was increased in a dose-dependent manner, up to 75% iPSC-CM (iPSC-CM:fresh basal medium = 3:1 mixture) but was reduced at 100%. It was supposed to be due to accumulation of toxic metabolites such as lactic acid and the depletion of nutrients in the iPSC-CM. For this reason, it was shown in the result of the MTT assay that the maximum increase in HDFs proliferation was limited, up to about 40% [34]. On the other hand, when iPSC-Exo was applied, the cell proliferation has increased by up to 80% without any significant cytotoxicity (Figure 2). Furthermore, the treatment of iPSC-Exo promoted the migration of HDFs similar with iPSC-CM. Although the rate of gap-filling may be affected by the proliferation as well as the migration in scratch wound assay (Figure 3A), the stimulatory effect of iPSC-Exo on the migration of HDFs was also clearly demonstrated in transwell migration assay (Figure 3B,C). These results indicate that iPSC-Exo contains key factors to induce the proliferation and migration of HDFs and is more suitable for clinical application than iPSC-CM because of its lower toxicity. There may be concern that nonexosomal proteins were contained in the isolated iPSC-Exo samples. However, it is likely that only a small fraction of the total nonexosomal proteins contained in the iPSC-CM has been precipitated with iPSC-Exo, and consequently, the concentration of the nonexosomal proteins was significantly lower when HDFs were treated with the isolated iPSC-Exo samples. It is also possible that some cytokines and growth factors among nonexosomal proteins were inactivated during the precipitation because they are known to be very labile (e.g., basic fibroblast growth factor). Taken together, it is unlikely that nonexosomal proteins exerted significant effects on the results shown in our study. However, a recent study revealed that ultracentrifugation method provided higher purity than ExoQuick^TM^ in exosome purification, although nonexosomal proteins such as serum albumin and apolipoprotein E were not completely removed even in this method [51]. They also suggested that a density gradient centrifugation is superior to protocols based on ultracentrifugation and precipitation in terms of purity, despite disadvantages such as high labor intensity. For further applications, we believe that it is necessary to develop an optimized iPSC-Exo purification protocol providing higher purity and exosome yield.

The present study also demonstrated the beneficial effects of iPSC-Exo on damage and alterations of gene expression induced by not only UVB irradiation but also natural senescence. UV in sunlight, consisting of UVA (320–400 nm), UVB (280–320 nm), and UVC (200–280 nm), is a major environmental factor in causing skin photoaging [38,52]. UVB results in cellular damage by inducing DNA mutations as well as indirectly via oxidative stress [53]. In addition, exposure of dermal fibroblasts to UVB activates cell surface receptors, which subsequently stimulate MAPK signaling pathways. MAPK signal transduction is associated with the transcription factor activator protein-1 (AP-1), which reduces the synthesis of collagen type 1 and enhances the expression of MMPs [3,38,39]. When iPSC-Exo was applied to HDFs, we found that cells were protected from damage induced by UVB irradiation. Since we have observed that iPSC-Exo promoted the proliferation of HDFs even without UV irradiation (Figure 2), the viability of each cell group was normalized by that of the UVB-unexposed group. Nevertheless, the protective effect of iPSC-Exo on UV-induced damage was clearly demonstrated (Figure 4). Meanwhile, we also found that the pretreatment of iPSC-Exo inhibited the upregulation of MMP-1 and MMP-3 and downregulated collagen type I mRNA expression induced by UVB irradiation (Figure 5). Next, the effects of iPSC-Exo on multiple genotypic changes involved in natural senescence were further demonstrated. The expression of SA-β-Gal, a typical senescence-associated marker [41], was significantly decreased by iPSC-Exo (Figure 6). Then, we found that iPSC-Exo restored altered expression of MMP-1, MMP-3, and collagen type I in senescent HDFs, as shown in the case of photoaging (Figure 7). These results suggest that iPSC-Exo contains useful factors to mediate the rebalancing process of the matrix in the aging skin and they can successfully be delivered into dermal fibroblasts. It is believed that those factors modulate the expression of aging-related genes and promote the reconstitution of dermal matrix by increasing the content of structural proteins such as collagen type I in aged skin.

Several studies have reported proteomic analysis of extracellular vesicles derived from various stem cells. Kim et al. profiled the proteome of microvesicles from MSCs (MSC-MVs) using mass spectrometry-based approach and identified 730 MV proteins. They identified potential MV protein candidates that can be implied in the therapeutic effect of MSC-MVs on damaged tissues via the integration of the MSC-MV proteome with the transcriptome of MSCs and the proteome of MSC-conditioned medium [54]. Anderson et al. identified 1927 proteins in MSCs-derived exosomes using high resolution isoelectric focusing liquid coupled chromatography tandem mass spectrometry (HiRIEF LC-MS/MS) and these proteins include several putative paracrine factors modulating angiogenesis [55]. Meanwhile, several proteomic analyses have identified numerous proteins, including cytokines, signaling molecules, chaperones, and plasma membrane proteins, from human pluripotent stem cell [56,57,58,59]. Similar to MSC-derived exosomes, iPSC-Exo is believed to contain many kinds of those human iPSC-derived proteins. iPSC-Exo may also contain various mRNAs, miRNAs, and lncRNAs, which can affect cellular physiologies. For the reason, the beneficial effects of iPSC-Exo on skin fibroblasts are presumably due to the complex action of various components such as cytokines, membrane proteins, and RNAs.

In conclusion, our results have revealed the beneficial effects of human iPSC-Exo on human skin damaged by photoaging and on natural senescence. To the best of our knowledge, this is the first study to investigate the effect of human pluripotent stem cell-derived exosomes on the treatment of aging skin. Exosomes are considered to have several advantages in that they can stably preserve useful components to prevent skin aging and efficiently deliver them to the skin tissues. Because many complicated processes are involved in skin aging, further study should be required to demonstrate the therapeutic potential of iPSC-Exo. However, the present study is expected to serve as a technical insight toward the clinical application of exosomes derived from human induced pluripotent stem cells for the treatment of skin aging.

## 4. Materials and Methods 

### 4.1. Cell Culture

In this study, we used a human iPSC line obtained from The National Center for Stem Cell and Regenerative Medicine in Korea. As described in our previous study [34], this cell line was generated from human dermal fibroblasts by introducing Oct4, Sox2, cMyc, and Klf4 using Sendai Virus. When the confluency was reached at 80%–90%, the iPSCs were dissociated by treatment with 0.5 mM EDTA and plated onto a truncated human vitronectin-coated culture dish. The cells were cultured in Essential 8 medium (Thermo Fisher Scientific, Waltham, MA, USA) and passaged every 4 to 5 days. In addition, human dermal fibroblasts (HDFs) were cultured in DMEM supplemented with 10% fetal bovine serum (FBS) and penicillin/streptomycin. To induce replicative senescence, HDFs were cultured for a long period of time with repeated passaging. When the confluency was reached at 70%–80%, HDFs were plated onto new dish at 2 × 10^4^ cells/cm^2^ and cultured until getting 70%–80% confluent again. All cells were cultured at 37 °C in a humidified atmosphere containing 5% CO_2_.

### 4.2. Exosome Isolation

Culture medium was replaced with fresh Essential 8 medium daily in human iPSCs culture. The spent medium was collected daily from day 2 to the end of culture and filtered through 0.45 μm syringe filter. Exosomes were isolated from the conditioned medium, using ExoQuick-TC^TM^ (System Biosciences, Palo Alto, CA, USA) according to the manufacturer’s instructions. Briefly, the conditioned medium was incubated with the exosome precipitation solution at 4 °C for 12 h and subsequently centrifuged at 1500× *g* for 30 min. After discarding the supernatant, the pellet was resuspended in phosphate buffered saline (PBS) and the collected iPSC-Exo was stored at −80 °C until use.

### 4.3. Characterization of iPSC-Exo

Size distribution and concentration of iPSC-Exo were analyzed by nanoparticle tracking analysis (NTA) using NanoSight NS300 (Malvern Panalytical, Malvern, UK). The solution containing iPSC-Exo was injected into the laser chamber using a 1 mL syringe and three 30 sec recordings were performed. The mode, mean size, and concentration of iPSC-Exo were determined by NTA 3.0 software. Camera level, threshold, and focus were kept under the same conditions in all experiments. Morphology of iPSC-Exo was visualized by transmission electron microscopy (TEM) using a JEM-1010 electron microscope (JEOL, Tokyo, Japan). First, iPSC-Exo was absorbed in a formvar/carbon-coated grid for 10 min and fixed with 2% paraformaldehyde. After negative staining with 2% uranyl acetate for 10 min, iPSC-Exo was observed by TEM operated at 60 kV. Zeta potential was measured using a Zetasizer Nano ZS (Malvern Panalytical, Malvern, UK).

### 4.4. MTT Assay

HDFs were plated at 2 × 10^4^ cells/cm^2^ in 96-well culture plates and incubated in growth medium for 24 h. For the serum starvation, the medium was then replaced with DMEM supplemented with 0.5% FBS and cells were cultured for a further 24 h. Following treatment of iPSC-Exo in serum-free DMEM/F12 for 24 h, MTT (3-(4,5-dimethylthiazol-2-yl)-2,5-diphenyltetrazolium bromide) solution (Sigma Aldrich, St. Louis, MO, USA) was added to each well and incubated for 4 h. After removal of the supernatant, dimethyl sulfoxide was added to dissolve formazan and subsequently the absorbance at 560 nm was determined using a microplate reader.

### 4.5. Migration Assay

The effect of iPSC-Exo on the migration of HDFs was determined by scratch wound assay and transwell migration assay as described in the previous study [34]. In scratch wound assay, briefly, a scratch was created by scoring 70%–80% confluent monolayer of serum-starved HDFs with a sterile pipette tip. After incubation with 20 × 10^8^ particles/mL iPSC-Exo in serum-free DMEM/F12 for 24 h or 48 h, migration of HDFs into the scratched area was monitored under an optical microscope. For the transwell migration assay, HDFs were plated onto upper chambers of transwell plates (Corning, Lowell, MA, USA) containing serum-containing growth medium and cultured for 24 h. Following replacement of the medium with serum-free DMEM/F12 containing iPSC-Exo, cells were incubated for a further 24 h to induce migration toward the opposite side of the transwell membrane. After removal of cells remaining on the top of the membrane by a cotton swab, the cells at the bottom of the membrane were fixed with 4% paraformaldehyde and then stained by 0.5% crystal violet (Sigma Aldrich, St. Louis, MO, USA) to visualize the migrated cells. The staining intensity was determined as absorbance at 560 nm after dissolving the crystal violet using 50% acetic acid.

### 4.6. UVB Irradiation

HDFs were plated in 96- or 24-well plates, depending on the assay, and cultured for 24 h. Then, cells were treated with 20 × 10^8^ particles/mL iPSC-Exo in serum-free DMEM/F12 for 24 h. After washing three times, 50 μL and 200 μL of PBS were added to 96- and 24-well plates, respectively. Cells were irradiated with UVB (315 nm) generated by a Bio-Sun illuminator (Vilber Lourmat, Eberhardzell, Germany). The light intensity was estimated according to the distance between the UV illuminator and the each well plate. After the UV irradiation, PBS was replaced with DMEM/F12 and the cells were further incubated for 24 h or 48 h prior to specific assays. For MTT assay, cells were divided into iPSC-Exo-untreated (Control) and treated group (iPSC-Exo). Each group was further divided into following three groups: UVB-unexposed, 40 mJ/cm^2^ UVB-exposed, and 80 mJ/cm^2^ UVB-exposed group.

### 4.7. SA-β-Gal Staining

Senescent HDFs were plated in 96-well plates and cultured for 24 h. Following 20 × 10^8^ particles/mL iPSC-Exo treatment for 24 h, the cells were further cultured for 48 h in serum-free DMEM/F12. The cells were then fixed with 4% paraformaldehyde and SA-β-Gal was stained using senescent cells histochemical staining kit (Sigma Aldrich, St. Louis, MO, USA). Three images per each well were collected, and the SA-β-Gal-stained cells were counted.

### 4.8. RNA Isolation and Real-Time RT-PCR

Total RNA was extracted from HDFs using a GeneAll Ribospin^TM^ total RNA purification kit (GeneAll Biotechnology Co. Ltd., Seoul, Korea) according to the manufacturer’s instructions. Purified total RNA was reverse-transcribed to cDNA using TOPscript^TM^ RT DryMIX (Enzynomics Co. Ltd., Daejeon, Korea) with a dT 18 plus primer. Subsequently, a PCR step was performed from the cDNA samples using specific primers and TOPreal^TM^ qPCR 2X PreMIX (SYBR Green with high ROX, Enzynomics Co. Ltd. Daejeon, Korea) in triplicate on the Eco Real-Time PCR System (Illumina, San Diego, CA, USA). The primers used in this study are summarized in Table 1. The standard cycle conditions were as follows: 95 °C for 1 min, 40 cycles of denaturation at 95 °C for 15 s, and annealing-extension at 60 °C for 30 s. The expression level of specific RNAs was normalized by actin as an endogenous control.

### 4.9. Statistical Analysis

All results were expressed as mean ± standard deviation and were statistically analyzed by a Student’s *t*-test. Statistical significance was indicated at *p* < 0.05.

## Figures and Tables

**Table 1:** List of primers for real-time RT-PCR.

Gene	Primer	Sequence (5′–3′)
β-actin	Sense	GTG GGG CGC CCC AGG CAC CAC
	Antisense	CTC CTT AAT GTC ACG CAC GAT TT
MMP-1	Sense	CAT CGT GTT GCA GCT CAT GA
	Antisense	ATG GGC TGG ACA GGA TTT TG
MMP-3	Sense	TGC TGC TCA TGA AAT TGG CC
	Antisense	TCA TCT TGA GAC AGG CGG AA
Collagen type I	Sense	CTC GAG GTG GAC ACC ACC CT
	Antisense	CAG CTG GAT GGC CAC ATC GG

## Abbreviations

iPSC	Induced pluripotent stem cell
HDF	Human dermal fibroblast
qPCR	Quantitative real-time polymerase chain reaction
MMP	Matrix metalloproteinase
PDGF	Platelet-derived growth factor
MAPK ECM	Mitogen-activated protein kinase Extracellular matrix
CM	Conditioned medium
TGF-β	Transforming growth factor-β
MSC	Mesenchymal stem cell
hDSPC	Human dermal stem/progenitor cell
UVB	Ultraviolet B
iPSC-CM	Induced pluripotent stem cell-derived conditioned medium
iPSC-Exo	Induced pluripotent stem cell-derived exosomes
SA- β-Gal	Senescence-associated β-galactosidase
NTA	Nanoparticle tracking analysis
TEM	Transmission electron microscopy
DLS	Dynamic light scattering
FBS	Fetal bovine serum
PBS	Phosphate buffered saline
MTT	3-(4,5-dimethylthiazol-2-yl)-2,5-diphenyltetrazolium bromide
lncRNA	Long non-coding RNA
